# Editorial: Exploring the emotional landscape: cutting-edge technologies for emotion assessment and elicitation

**DOI:** 10.3389/fpsyg.2024.1542158

**Published:** 2025-01-07

**Authors:** Ivonne Angelica Castiblanco Jimenez, Federica Marcolin, Enrico Vezzetti, Javier Marín Morales, Alessia Celeghin

**Affiliations:** ^1^Department of Psychology, Università degli Studi di Torino, Turin, Italy; ^2^Department of Management and Production Engineering, Politecnico di Torino, Turin, Italy; ^3^Institute for Human-Centered Technology Research (HUMAN-Tech), Universitat Politècnica de València, Valencia, Spain

**Keywords:** emotion assessment, affective elicitation, biometrics, physiological data, neuroscience, affective computing, augmented/virtual reality, human-computer interaction

## The challenge of capturing human emotions

The assessment of human emotions in psychological research presents multiple methodological challenges. While traditional measurement approaches have contributed valuable insights, they often face limitations in capturing the complexity of emotional experiences and maintaining ecological validity. Recent technological advances provide new opportunities to address these challenges, enabling more precise and naturalistic approaches to emotion assessment and elicitation.

This Research Topic reunites research that employs emerging technologies for emotion research. The collected works demonstrate how various methodological approaches can overcome traditional limitations while expanding our understanding of emotional processes in both controlled and natural settings. These advances have been demonstrated in various novel technologies applications that are redefining how we study and understand emotions. From virtual reality environments to artificial intelligence and innovative physiological measurements, these progresses are expanding the landscape of emotion research.

## Technological advances in emotion assessment

Virtual reality technology has emerged as a powerful tool for psychological research and therapy. Zhang et al. present a systematic investigation of the Apple Vision Pro's capabilities in emotional research and therapeutic applications. Through the integration of multi-sensor technology, high-resolution displays, and remote meeting capabilities, their work highlights VR's potential for creating immersive environments suitable for both emotion assessment and therapeutic interventions.

The integration of artificial intelligence with neurophysiological data represents another significant advancement in emotion research. Wang et al. present an innovative approach using vision transformers for EEG-based emotion classification during music listening tasks. Through advanced neural network architectures, their work advances automated emotion recognition from brain activity patterns.

In a complementary investigation of physiological intervention methods, Cong et al. demonstrate how transcutaneous electrical acupoint stimulation at PC6 can effectively reduce fear and improve emotion regulation, as evidenced through changes in heart rate variability.

The complexity of emotional experiences demands sophisticated measurement approaches. In learning environments, Horvers et al. advance this field by investigating emotional responses to immediate feedback during math problem-solving tasks. Their work combines physiological signals (electrodermal activity, electrocardiogram) with experiential and behavioral measures (self-reports, observations of facial expressions) to provide richer insights into emotional experiences, highlighting the value of multimodal assessment approaches in educational settings.

Emerging research further establishes concrete metrics for emotion detection in learning environments. Lal et al. identify specific physiological and behavioral indicators of learner emotions through careful analysis of skin conductance, temperature, and eye movements. Their findings reveal that measures such as skin conductance response peaks and eye-tracking metrics can effectively distinguish between emotional states, offering practical tools for emotion-aware learning technologies.

The challenge of collecting emotion data in natural settings has led to innovative solutions. Niewiadomski et al. present a framework for physiological data collection based on appraisal theories. Using a wearable device to collect physiological signals (blood volume pulse, electrodermal activity, skin temperature) and movement data, their mobile application detects potentially relevant emotional events and prompts users for self-reports, bridging the gap between laboratory precision and real-world applications.

The effectiveness of emotion regulation strategies varies significantly across different contexts and individuals. Int-Veen et al. examine this complexity through their study of daily emotional regulation practices, revealing important relationships between regulation strategies, self-efficacy, and stress management. Their findings highlight how different regulation approaches may be more or less effective depending on individual characteristics and situational factors.

Physical environments play a crucial role in shaping emotional experiences. Zhang analyses students' emotional attachment to a renovated campus landscape space called the “Heart of Forest,” a 12,000-square-meter public activity area at Beijing Forestry University. Through a mixed-method approach combining emotional attachment scales with sentiment analysis, this study reveals how landscape design can enhance students' emotional attachment and wellbeing in educational settings.

## Challenges and opportunities in emotion research

The multimodal approaches presented in this Topic highlight specific challenges in data harmonization and interpretation. The integration of EEG measurements, physiological interventions, and eye-tracking metrics requires careful consideration of different temporal and spatial scales. Applications in educational and therapeutic settings illustrate the need to balance technical capabilities with practical implementation. Furthermore, as monitoring extends into daily-life contexts, questions of privacy and informed consent become central to research design and implementation.

This Research Topic demonstrates significant progress in emotion research methodology through technological innovation. Each study advances our understanding while highlighting the importance of maintaining methodological rigor in real-world applications. The convergence of virtual reality, physiological sensing, and artificial intelligence creates new possibilities for understanding emotional processes. As these technologies continue to develop, future research must address both the technical challenges of data integration and the ethical considerations of continuous emotional monitoring. This field moves forward not just through technological advancement, but through careful consideration of how these tools can best serve our understanding of human emotional experience.

